# Community-led initiatives for the rehabilitation and management of vernacular settlements in Oman: a phenomenon in the making

**DOI:** 10.1186/s43238-021-00039-5

**Published:** 2021-12-14

**Authors:** Benkari Naima

**Affiliations:** Department of Civil and Architectural Engineering, Sultan Qaboos University, P.O. Box 33, P.C. 123, Al-Khoudh, Muscat, Sultanate of Oman

**Keywords:** Adaptive reuse, Community involvement in heritage management, Gulf cooperation council (GCC) states, Heritage houses, Heritage tourism, Post-oil economy, Sustainable development, Vernacular settlements

## Abstract

Once abandoned for more than three decades, vernacular settlements in Oman are now being progressively reinvested in to foster the country’s heritage tourism sector. The present research focuses on the emerging phenomenon of community-led initiatives for vernacular heritage rehabilitation and adaptive reuse in Oman. Through an examination of three case studies, its aim is to describe this process and its modes of action and discuss its effects on vernacular settlement transformations. A mixed research methodology was designed to include (A) analyses of relevant primary and secondary data, (B) documented onsite observations, (C) interviews with local community representatives and key players in the operations of rehabilitation, and (D) extractions and analyses of quantitative data from a hotel booking website.

The research sheds light on unsuspected interrelations within and between the projects being implemented in these settlements and their operating modes. It reveals the focal role of a local community in a kind of ‘bottom-up’ management of its built heritage, coupled with a ‘horizontal cooperation’ between the three initiatives studied in this research. Moreover, it shows that a heavily centralised and top-down policy for the field of heritage conservation and management is among the main obstacles that hinder such initiatives. Furthermore, community-led operations of vernacular heritage rehabilitation are being undertaken under insufficient regulations in terms of land use, building restoration and adaptive reuse. In this context, the paper discusses some of the serious threats and concerns faced by such initiatives and proposes actionable solutions to mitigate these hindrances.

## Introduction: vernacular heritage in Oman

Socioeconomic changes that began during the 1970s in Oman have led the government and local populations to adopt different approaches towards built heritage and its integration with the development process of the country. Considered one of the bases on which the Omani nation’s identity has been constructed (Klinger 2020, 502; Sachedina 2016; Nutz 2013; Bandyopadhyay 2005b, 19), Oman’s built heritage was often presented through an abstracted image of its defensive structures and military monuments (such as forts, towers and fortified palaces). A consistently formulated narrative about a unified Omani people with a millenary history was progressively built through a long process of the de-contextualisation and occasional ‘over restoration’ of carefully selected monuments (Klinger 2020; Sachedina 2016; Bandyopadhyay 2005a, 34). Except for some prominent mosques and civilian structures, the rest of Oman’s built heritage did not receive the same attention or play the same role in this narrative. Regarded as a ‘minor heritage’, vernacular settlements and their traditional stone and earthen architecture were superficially addressed by laws and mostly neglected by both the government and the community (Bandyopadhyay 2000; Benkari 2017b; Benkari, Naima, Salim Woaud Al-Araimi, and Khalifa Khalsa Al-Salmi: Oman heritage policy and its applications in the built heritage protection, under press). Such a situation is not unique to Oman, as vernacular heritage was only recently recognised as ‘worthy’ of consideration and protection (De Filippi 2005, 4,5; Salazar 2012; ICOMOS 1999; Correia et al. 2014, 171).

Since the late 1960s, the majority of the vernacular settlements in Oman were progressively abandoned in the aftermath of oil discoveries and exploitation (Benkari 2017c, 178; Al-Abrī, Haitham Najeem Sulaiman: Urban pattern and architecture of traditional Omani foothill settlements: Al-Ḥamrā and Birkat Al-Mawz, unpublished thesis, 159; Klinger 2020, 126, 544). Enjoying high revenues, most Omanis deserted their vernacular houses, which they deemed unsustainable for modern standards of comfortable living, such as running water, electricity and air conditioning. Instead, they built ‘modern villas’ out of cement in the main cities or in settlements near their land of origin. Today, this vernacular heritage is in an advanced state of decay due to weathering and as a result of abandonment and neglect. A few of these dwellings have been reused by foreign workers, brought to Oman to perform manual labour, which Omanis have progressively deserted, in agriculture, animal husbandry, and other occupations in the fields of craftsmanship and construction (Benkari 2017c; Al Shueili 2015, 171; Klinger 2020, 111, 121, 166).

Anticipating the dawn of a post-oil era, which has been foreshadowed for the past two decades through successive crashes in oil prices, Oman had to seriously consider diversifying its economy, which is heavily reliant on oil exports. The updated Oman tourism strategy 2040 clearly states that the country will consider tourism one of the main pillars of its economic policy by the third decade in this millennium (Ministry of Tourism 2016, 37–38). As a tool for the implementation of this strategy, the National Program for Enhancing Economic Diversification (TANFEEDH) recognizes built heritage has a solid potential for promoting the country’s identity and fostering the diversification of its economy, notably through heritage tourism (Tanfeedh Handbook 2017, 31, 35). In addition, an abrogation of the Heritage Law 06/1980 by the ‘New Heritage Law’ (Royal decree No. 75/ 2020) and a subsequent merger of the Ministry of Tourism and the Ministry of Heritage & Culture into the Ministry of Heritage & Tourism (Royal Decree No. 92/ 2020) could be interpreted as strong steps towards the implementation of Oman’s 2040 vision (Benkari, Naima, Salim Woaud Al-Araimi, and Khalifa Khalsa Al-Salmi: Oman heritage policy and its applications in the built heritage protection, under press). Furthermore, important companies have been created by the government with shared funds between public and private investors. ‘Omran’^1^ and ‘Asaas’^2^ are among the companies involved in real estate development through heritage, including old settlement renovations and management.

Some regions in the country with a distinguished vernacular heritage have regained the interest of local communities due to their high potential for developing low-density tourism that is centred on their landscapes (Buerkert et al. 2010) and heritage. The interior region (A-Dakhiliyah) seems to be the most coveted by these community-led operations. Therefore, such transformations in the approach to vernacular heritage in Oman and their methods of operation merit investigation and discussion from the particular perspective of the role of local communities in these processes.

The present research focuses on the emerging phenomenon of community-led initiatives of vernacular heritage rehabilitation and adaptive reuse regarding heritage tourism in Oman. Through an examination of three case studies, its aim is to describe this emerging phenomenon and its mechanisms of action to discuss its effects on vernacular settlement regeneration and transformation. The following research questions direct this study:

1. What is the legal framework for the protection, restoration and management of built heritage in Oman? How is it implemented?
2. What are the dynamics that govern the recent operations of rehabilitation and adaptive reuse of vernacular heritage in Omani settlements, and what are the roles of local communities in such operations?
3. What are the possible threats to such operations, and what are the appropriate solutions to alleviate them?
4. What are the effects of these operations on the regeneration and transformation of these traditional settlements?

## Built heritage management: from the top-down approach to community participation

Like several other countries, Oman has a predominantly ‘top-down’ built heritage management system. This approach has been questioned relatively recently (De Filippi 2005; Salazar 2012; Davis et al. 2010; El-Borombaly and Molina-Prieto 2015; Lenik 2013; Klinger 2020, 112, 484), but the situation is still unchanged in Gulf Cooperation Council (GCC) states. Research has shown that a heavily centralised, top-down management system is one of the main hindrances to optimal investment in vernacular heritage for urban and economic development (De Filippi 2005; Lenik 2013; Salazar 2012; Davis et al. 2010). It has also revealed the problems inherent to the top-down approach in these interventions, such as high costs (De Filippi 2005; Salazar 2012; Davis et al. 2010, 9), ‘*museification*’ of heritage areas (De Filippi 2005), and non-adherences by inhabitants, who tend to abandon these areas once the work of international restoration experts is complete (De Filippi 2005; Salazar 2012).

Consequently, researchers and professionals in this field have been calling for the increased awareness and engagement of local communities in the process of decision making related to their built heritage management and development plans (De Filippi 2005, 4–5; Lenik 2013; Chirikure and Pwiti 2008; Salazar 2012; Rivière 1985; Klinger 2020, 266). These calls have been supported and relayed by international bodies, such as the United Nations and the United Nations Educational, Scientific and Cultural Organisation (UNESCO), which have proposed a bottom-up approach to heritage management, coupled with existing top-down mechanisms (De Filippi 2005, 5; Davis et al. 2010, 8; Correia et al. 2014, 162,164; Brundtland 1987; ICOMOS Florence Declaration 2014). Although community engagement has evolved to characterize contemporary heritage practices within the field of archaeology (Tlili 2008; Waterton 2005; Crooke 2007; Smith 2006; Smith and Waterton 2013), in policy development, resource management (Watson and Waterton 2010, 2; Chirikure and Pwiti 2008), tourism development (Watson and Waterton 2010, 2), and some building restorations (such as mosques) (Klinger 2020, 231), community engagement remains relatively lacking, particularly in the field of vernacular heritage restoration and management. In Oman and some other GCC states, the approach is still in its embryonic stage. These countries offer a context in which financial resources are relatively available, professional skills and techniques are extremely limited, and relevant policies are frail and non-comprehensive (Al-Belushi 2014, 2015). Consequently, over the past 20 years, Oman and the other GCC states have displayed very little interest in developing their ‘heritage industry’, notably through tourism. Since the turn of the new millennium, awareness of the economic value of their heritage has notably increased amongst the Omanis and their government (Benkari 2018; Ministry of Tourism 2016; Klinger 2020, 258, 391). The recent successive oil crises and the subsequent economic recessions have swayed the country to finally adopt a nationwide plan to integrate cultural heritage with economic policy.

The present research addresses community involvement regarding built heritage management in Oman. It sheds some light on an emerging form of ‘community-led investment projects in Heritage’, where a local community is the initiator of and main investor in such projects. At this stage, it is important to follow Salazar, Watson and Waterton and others; the concept of ‘community’ should be grasped by its transformative meaning. This meaning includes all actors and members affected and concerned by a vernacular heritage (Waterton 2005; Crooke 2007; Tlili 2008; Smith and Waterton 2013; Smith 2006; Salazar 2012, 10; Watson and Waterton 2010).

A review of the scientific literature focused on Omani vernacular heritage showed that since the 1970s, this topic has interested scholars from different disciplines, such as archaeology, anthropology, social studies, history and architecture. However, though the legal framework related to the protection and management of this heritage has been questioned (Al-Belushi 2014, 2013; Benkari, Naima, Salim Woaud Al-Araimi, and Khalifa Khalsa Al-Salmi: Oman heritage policy and its applications in the built heritage protection, under press), the specific aspects of community involvement in its rehabilitation and reuse have yet to be investigated (Benkari 2019). The present article is, therefore, a timely discussion of this question within the context of three case studies of community-initiated, vernacular rehabilitations of houses for cultural tourism. The aim is to analyse such operations at the local level within the socioeconomic context of the country. To achieve this goal, the paper discusses the inception and development of each operation, highlights the continuities and differences between the three cases and examines their potentials and challenges. Finally, it concludes with a summary of the lessons learned and offers some insights into the possible options to further stimulate and guide community-led vernacular heritage management in Oman.

## Methodology

To address this study’s research questions, a mixed research strategy was designed to include (A) analyses of relevant primary and secondary data, (B) documented onsite observations, (C) interviews with local community representatives and key players in the operations of rehabilitation, and (D) extractions and analyses of quantitative data from a hotel booking website. The investigation was initiated in 2015 with ‘al-Misfat guest house’, one of the first local community-led operations of heritage management for touristic purposes in the governorate of A-Dakhiliyah in Oman (Benkari, Naima, and Alya Al-Hashim: Omani traditional houses and settlements: a new options for developing a sustainable tourism, unpublished). Two other case studies were included in successive stages after the opening of their guest houses or hotels in 2017 and 2018. Both primary and secondary data were used to address the previously mentioned questions.

An extensive literature review included the study of scientific and general publications, official documents, and other brochures and promotional materials. The review facilitated an identification of the state of knowledge regarding the research subject and the development of a theoretical framework. The primary data were collected during multiple site visits, undertaken yearly since 2015. Through photographic and video recordings, these visits aimed to document the transformations in the three settlements resulting from rehabilitation and restoration operations. Such documentation was intended to provide a deeper understanding of the emerging phenomena (Salazar 2012, 13–14; Noussia 2003, 200–201).

Semi structured interviews were carried out with key actors in the initiatives of rehabilitation and restoration, as well as with some randomly selected local residents and tourists staying in newly opened guest houses. The research aim was to study the inception and progress of initiatives regarding the restoration of traditional houses for their adaptive reuse for touristic functions. Therefore, the interviews targeted key actors who held leading positions in a group managing one of the projects. Due to the time limitations for this research and the limited availability of such participants for the interviews, a minimum of one interviewee per case study was targeted. The reliability of the information received was assessed, whenever possible, through comparison with a second or third interviewee (Patton 1999; Denzin 2017; Flick 2004). Given that the initiatives started and progressed according to different timelines, the interviews took place in different years, based on the level of maturity of each operation and the availability of its initiators to meet the researcher and share their experiences. The first interviews took place in 2016 in Misfat al ‘Abriyin, with a member of the family who started the project to restore and adaptively reuse its own houses. In April 2018, two interviews were conducted in Harat al-Hamra, the second case study. The first interview was with the former president of the association of local residents and owners, who initiated the project to restore houses in al-Hamra. The second interviewee was the actual president of the association, who was also the contractor in charge of the restoration works and the director of the first guest house in al-Hamra. Later, in the same year (July 2018), interviews were conducted in Harat al ‘Aqr, the third case study, with the general manager and a board member of the private investment company that initiated and continues to manage restorations of decaying houses in Al-‘Aqr. A second board member, a director of one of the recent heritage hotels inaugurated in the same settlement, was interviewed in February 2021.

The leading questions in the interviews were designed to guide an interviewee to narrate the initiative, including the inception, of a project, as well as its planning and progress. Some questions were also intended to address the main obstacles encountered during the different stages of an operation and the strategies used to overcome them. All interviews were performed in Arabic with their video or audio recorded through the prior consent of the interviewee and none lasted more than one hour. The content was then transcribed, categorised and analysed to extract the relevant direct information from each case study and to identify key themes and patterns across all three cases (Bryman 2016; Kvale and Brinkmann 2009). It was not possible to interview government representatives of A-Dakhiliyah region (where the case studies are located) due to the complex administrative procedures required. To overcome this limitation, timely questions related to the research were conveyed to representatives of the government in other regions to obtain their feedback and opinions about these initiatives.

Respecting confidentiality policies regarding tourists staying in the restored properties made it impossible to collect useful tourist data directly from the interviewees. Therefore, without being a core source for data collection, a quantitative approach was used, but only to provide an approximate figure for the level of tourist frequency in the first case investigated (al-Misfat guest house). A website, booking.com, was selected to collect information regarding the number of visitors and visitation periods. Booking.com is a well-established hotel booking website, from which it is possible to extract open access information about hotel guests, their country of origin and the period and length of their stays in a given hotel, as well as their comments about a hotel and its amenities. Such websites are now an established source of quantitative data, often used by researchers in the field of tourism and hotel management (Díaz and Espino-Rodríguez 2018). Booking.com was selected specifically because it is among the most used websites in this field; hence, it contained the highest number of tourists’ comments about ‘al-Misfat guest house’, which was the first to be listed on this website. The numbers illustrated in Fig. 6 were deduced from the number of comments recorded on the page for al-Misfat guest house on Booking.com. All guests who left a comment between August 2018 and March 2021 were recorded on an MS Excel spreadsheet according to their date of visit, country of origin, language and category (solo, couple, family or group of friends). To simplify counting the guests, each recorded comment (C) was given a numerical value (N) that corresponds to the type of category, indicating the number of visitors. Therefore, *N* = Cx1 for a solo traveller, *N* = Cx2 for a couple, *N*=Cx3 for a group of friends, and families were assumed to be *N* = Cx3.5 (Table 1). The numbers thus generated may not reflect the exact rate of frequency of a property by tourists, but they can be used to clearly estimate its annual progress of use.

**Table 1:** Method of estimation for the number of guests in Al-Misfat guest houses

Category of visitors	Number of comments by this category (C)	Calculated number of persons in each category (N)	Formula
Number couples	309.00	618	*N* = Cx2
Number family	139.00	486.5	*N* = Cx3.5
Number group	105.00	315	*N* = Cx3
Number solo	45.00	45.00	*N* = Cx1
**Total reviews**	**598**	**1464.5**	**Total people**

Finally, updates about the studied properties were regularly collected through an established network of local informants. This included people from the communities involved in heritage projects, contacts in Oman’s Ministry of Heritage and Tourism, and from the author’s actual or graduated students residing or working in the studied areas.

## Results

The present research discusses three case studies of community-led initiatives for the restoration and rehabilitation of vernacular dwellings and their adaptive reuse for heritage tourism. It is articulated around the following questions:

1. What is the legal framework for the protection, restoration and management of built heritage in Oman? How is it implemented?
2. What are the dynamics that govern the recent operations of rehabilitation and adaptive reuse of vernacular heritage in Omani settlements, and what are the roles of local communities in such operations?
3. What are the possible threats to such operations, and what are the appropriate solutions to alleviate them?
4. What are the effects of these operations on the regeneration and transformation of these traditional settlements?

### Built heritage in Oman: its protection and management

**Fig. 1:**
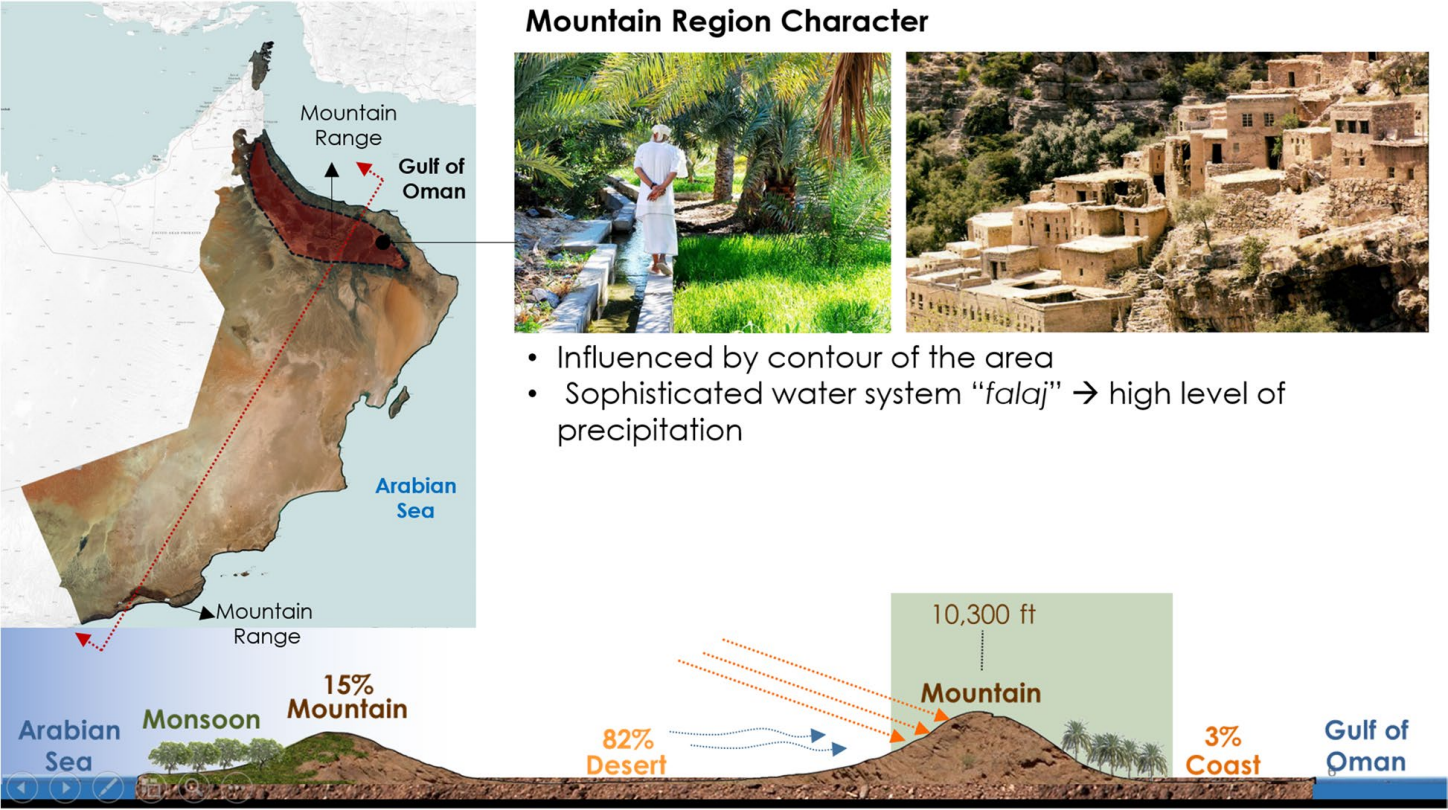
Geographic layout of Oman, highlighting the mountainous region (Source: Benkari, Naima, and Alya Al-Hashim: Omani traditional houses and settlements: a new options for developing a sustainable tourism, unpublished)

The Sultanate of Oman is composed of four main geographical regions: the coastal region in the east; the mountainous chains; the desert, which is part of the Empty Quarter; and the monsoon region, where a seasonal, equatorial climate attracts both domestic and international tourism to the region during the summer (Khareef). Vernacular architecture in Oman varies according to the geographic and climatic character of each region (Al-Hinai et al. 1993) (Fig. 1).

**Fig. 2:**
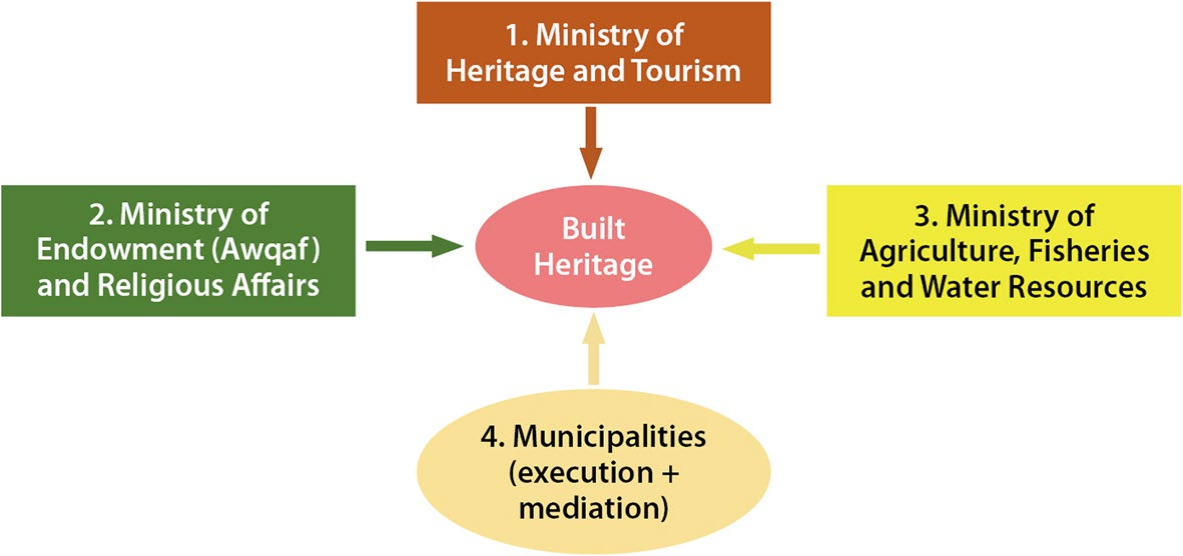
The bodies managing built heritage in Oman (Source: The author based on Royal Decree 75/ 2020)

The Ministry of Heritage and Tourism 6059 monuments registered in Oman: 1029 historical settlements (Harats), 1172 watch towers, 364 forts, 347 historical mosques, 136 castles, 53 historical walls, 132 heritage houses (8 of which are private) and 2826 underwater artefacts (National Centre for Statistics and Information 2020, 8, 17). Oman is also rich in more than 4000 falaj^3^ (Al-Ghafri 2018), most of which date back to the 17th and 18th centuries, and 3108 of which are still functioning (Al-Hatmi and Al-Amri 2000). This heritage attracts an increasing number of tourists who travel to visit these places and sites despite Oman’s insufficient touristic infrastructure compared to neighbouring GCC states (National Centre for Statistics and Information 2020, 19). A 2018 study revealed that both Omanis and residents are aware of the importance of this heritage for fostering Oman’s national identity and economy (Benkari 2018). Nevertheless, this important asset has suffered from frail management systems (Al-Belushi 2014) and responsibilities scattered among several ministries and public authorities (Benkari, Naima, Salim Woaud Al-Araimi, and Khalifa Khalsa Al-Salmi: Oman heritage policy and its applications in the built heritage protection, under press). Nevertheless, a recent remodelling of the governmental system under the newly enthroned Sultan Haitham Bin Tariq Al-Said merged the Ministry of Heritage and Culture with the Ministry of Tourism to create the Ministry of Heritage and Tourism (MHT) (Royal decree No. 75/ 2020). Today, the MHT is the main body managing built heritage in Oman. It has also integrated the responsibilities of the dissolved Public Authority for Craft Industries. The Ministry of Endowment (Awqaf) and Religious Affairs still oversees the management of historical mosques, but their restoration and maintenance is now the responsibility of the MHT. The Ministry of Agriculture, Fisheries & Water Resources is responsible only for the maintenance and development of the falaj systems. Additionally, the Office of His Majesty’s Advisor for Cultural Affairs, which previously managed some archaeological sites, was dissolved, and the MHT was given authority over all sites. However, municipalities in different governorates do not have any direct authority regarding the management or protection of built heritage. Their responsibilities are limited to the adherence and transmission of MHT decisions to local communities (Fig. 2).

Heritage protection and management in Oman is a top-down process; decisions are centralised, in Muscat, at the level of the concerned ministries, mainly the MHT. They are then transmitted to administrative offices in the governorates, which relay them to local municipalities, the walis (governors) and local community representatives to be executed. Such has been the situation since the emergence of Oman’s nation-state in 1970, under the rule of the late Sultan Qaboos B. Said, when the political system was changed from a traditional system of governance. The power of tribes and local communities, which was much more important before the 1970s, has been gradually replaced by a rather centralised governing system, whereby local communities have mostly been excluded from the management of their built heritage (Klinger 2020, 205; Scholz 2018).

#### Vernacular settlements in a modernised Oman

Before its modern era, Omani society had a traditional organisation that was sufficient to manage its political, religious and socioeconomic activities, such as going to war, digging a new falaj, initiating a new settlement or erecting a fortification. Many initiatives were undertaken by local communities following approval by the ruler of the country (sultan or imam). The late 1960s were marked by the discovery and subsequent trade of oil. This was closely followed by the ascension to the sultanate by the late Sultan Qaboos Bin Said Al Said in 1970. These circumstances triggered an unprecedented modernisation in major aspects of Omani life, namely, transportation, built environments, education, health and welfare. The national economy underwent a rapid transformation, shifting away from coastal areas’ reliance on fishing or commerce and mountainous regions’ dependence on agriculture and traditional crafts. Both areas were morphed into an economy exclusively based on the exportation of oil and its by-products, akin to other GCC states. All amenities, ministries and services have since been centralised in Muscat, the capital city (Benkari 2017a; Klinger 2020, 235, 391). Thus, working-age Omanis were attracted to the capital and progressively abandoned the traditional settlements of their ancestors. Despite Omanis’ famous weekly exoduses or seasonal moves from the capital and the main cities to their birthplaces, most of the traditional settlements are largely deserted today. Even people who do not leave their birthplaces tend not to live in the traditional dwellings of their ancestors because they do not satisfy their aspirations for a ‘modern’ lifestyle and its particular requirements regarding thermal comfort. Rather, they prefer to build new air-conditioned cement houses with more rooms and wider windows, often across the road from an original settlement.

Because of this process of abandonment, many settlements and their houses are now in an advanced state of decay. Today, deserted houses with falling roofs and crumbling walls, bordered by palm oases, banana and mango groves, irrigated by long water channels which often pass between the ruins, are a common landscape that reflects the state of conservation in the traditional settlements in Oman. A few dwellings in some settlements are occupied by low-income foreign workers, mainly from Southeast Asia, employed in palm tree plantations, for falaj maintenance or through other local crafts. These new inhabitants have different uses for the old earthen houses and cannot suitably maintain them. Hence, these occupants have become a factor that accelerates the decay of these buildings by altering their architectural elements, adding rooms and new equipment, such as electrical installations, HVAC and plumbing systems, with no consideration of whether the ancient structures can withstand these changes (Benkari 2021). Disruptive transformations such as these are recent, though common occurrences in abandoned settlements. Their effect is also limited compared to erosion through the neglect of the vernacular dwellings by their owners, despite the strong emotional attachment many Omanis have to their built heritage (Benkari 2018).

#### Vernacular architectural heritage restoration in Oman

Vernacular built heritage was lightly addressed in the NHPL (6/1980) and in the Cultural Heritage Law (35/2019). However, the MHT has recently invested a serious and commendable effort in the documentation of vernacular heritage, especially for the most endangered settlements, their dwellings and other structures. The extensive urban and architectural surveys conducted, in this context, by the Centre for the Study of Architecture and Cultural Heritage of India, Arabia and the Maghreb (ArCHIAM) and the studies prepared by the Sultan Qaboos University (Benkari 2021), are the fruits of this effort. In terms of the actual rehabilitation operations, the actions have not been as extensive or systematic. Only public buildings categorised as ‘national heritage’, such as forts, castles, towers, fortified palaces, royal or tribal mansions or important ancient mosques, may be restored using public funds (Hegazy 2015; Benkari, Naima, Salim Woaud Al-Araimi, and Khalifa Khalsa Al-Salmi: Oman heritage policy and its applications in the built heritage protection, under press). Notably, the role of the MHT in the restoration of privately owned properties (especially houses) is better defined in the new Cultural Heritage Law (35/2019).

Additionally, vernacular settlements were also superficially addressed by the legislation. The only exception is the Oasis of Bahla, which, along with its fort (Bahla Fort), merited a conservation and management plan in 2019; more than three decades after it was inscribed in the World Heritage List in 1987 (Ministerial Decision No. 81/ 2019). Similar settlements have not been considered among the restoration operations undertaken by the government, except for Harat Al Bilad in Manah (Bandyopadhyay 2004, 2005a; Hegazy 2014; Bandyopadhyay 2000). Although completely deserted since the late 1970s, Harat Al Bilad was acquired by the state, and the owners were compensated as per the relevant legal procedures (Royal Decree No. 6/ 1980). However, only some of its structures were restored; mainly those located along its main spine. Management of the whole settlement was delegated to a cultural investment company (Omran) as per the ministerial decisions related to the Executive Regulations of Tourism. An entrance fee was also set, akin to all visitable museums affiliated with the Ministry of Heritage and Culture (Ministerial Decision 39/ 2016; Ministerial Decision 136/ 2016). Subsequently, due to its weak management, the settlement did not attract the anticipated number of visitors. Hence, it was locked down immediately after the outbreak of COVID-19, once again leaving its beautifully decorated mosques and dwellings to decay. Given the good intentions that were behind this project and despite the vast amounts of money dedicated to its realisation, the restoration of Harat Al Bilad represents yet another case that demonstrates the negative effects of a top-down approach to the rehabilitation and reuse of heritage buildings and settlements in Oman.

On the other hand, some privately funded initiatives or house restorations have been recorded over the past decade in different locations with varying levels of importance, via diverse funding sources. Bait Al Zubair (Klinger 2020) or Bait Al Khalili in Muscat, Bait Al Ghasham in Nakhal, and Bait Al-Sail Al-Ghassani in Salala are relatively prominent examples of those initiatives. The costs of traditional construction materials (stone and sarooj^4^) and the wages of the specialised contractors and skilled builders required for restoration and conservation cannot be easily met by middle-class families or even regional municipalities. Therefore, local communities tend to restore, refurbish, and rebuild the houses in their vernacular settlements with materials and techniques that are both affordable and readily available. This practice has become known as the ‘business as usual’ approach of the old houses’ restoration and repairs. Usually, an entire operation is funded by the owners or the people to whom they have rented their houses.^5^ Such operations have multiplied and spread over the past few years due to thriving cultural tourism in Oman (National Centre for Statistics and Information 2020) and an absence of regulations regarding vernacular architecture restoration and traditional settlement regeneration.

### Case studies

**Fig. 3:**
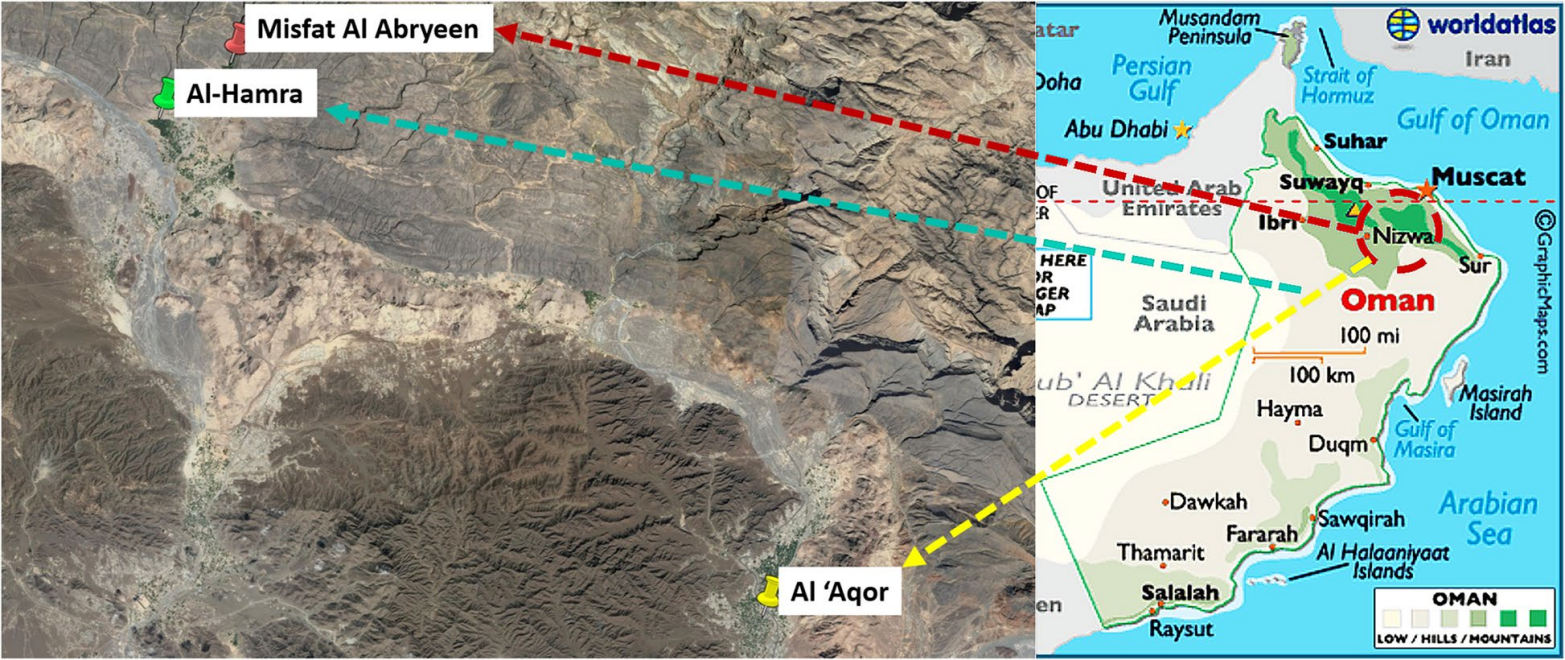
Location of the three case studies in the region of A-Dakhiliyah (Source: Google Earth image enhanced by the author)

The cases studied in this research are private houses located in Misfat Al-Abriyin, Al-Hamra and Al ‘Aqor. These three settlements are situated in the governorate of A-Dakhiliyah, not far from the famous city of Nizwa, one of the ancient capitals of the country. They are located at different altitudes: Al ‘Aqor is near the level of the city and its famous fort, Al-Hamra extends to slightly higher on the slope, and Misfat al-Abriyin is nested in a mountainous valley at an altitude of 950 m (above sea level) (Fig. 3).

The latter is the most populated and, hence, the best preserved of the three case studies. Al ‘Aqor and Al-Hamra have some houses that have been deserted or are inhabited by foreign workers and are in an advanced state of decay. Certain other houses have been rebuilt in cement, and some of them are still occupied by their original owners or their descendants.

#### Misfat Al-Abriyin

Both Misfat Al-Abriyin and Harat Al-Hamra are part of the territory of the Al-Abriyin tribe’s ‘dar’ (capital) in Wilayat Al-Hamra. Located approximately 200 km southwest of Muscat, this territory extends from the lower hills behind Jabal Shams to the northwest borders of Jabal Al-Akhdar. It is surrounded by low hills in which soils and rocks tend to have reddish shades and to which this region owes its name: ‘Al-Hamra (i.e., the red)’ (Al-Abrī, Haitham Najeem Sulaiman: Urban pattern and architecture of traditional Omani foothill settlements: Al-Ḥamrā and Birkat Al-Mawz, unpublished thesis, 66–69; Adawi 2006, 14). Based on a local story about the remains of the fort that dominates Misfat Al-Abriyin (Hosn Rogan), it is possible that the origins of this settlement date back to the Sassanid Era (Al Adawi 2006, 59), but no material evidence has been found to support this (Gaube 2014; Bandyopadhyay and Quattrone 2016). Nevertheless, the date of 1653 can clearly be seen, carved on the wooden ceiling of one of the most prominent dwellings in the core of the settlement.^6^ Similarly, the documents related to the falaj that irrigates this settlement’s oases date it to the mid-17th century during the reign of the Al Ya’rubi Imamate (Bandyopadhyay and Quattrone 2016, 24–25). The settlement’s strategic location, a strangely intricate setting within the mountain’s folds and picturesque landscapes, entails that Misfat Al-Abriyin has been featured in numerous published studies (Damluji 1998; Gaube 2014; Nagieb et al. 2004; Bandyopadhyay and Quattrone 2016).

**Fig. 4:**
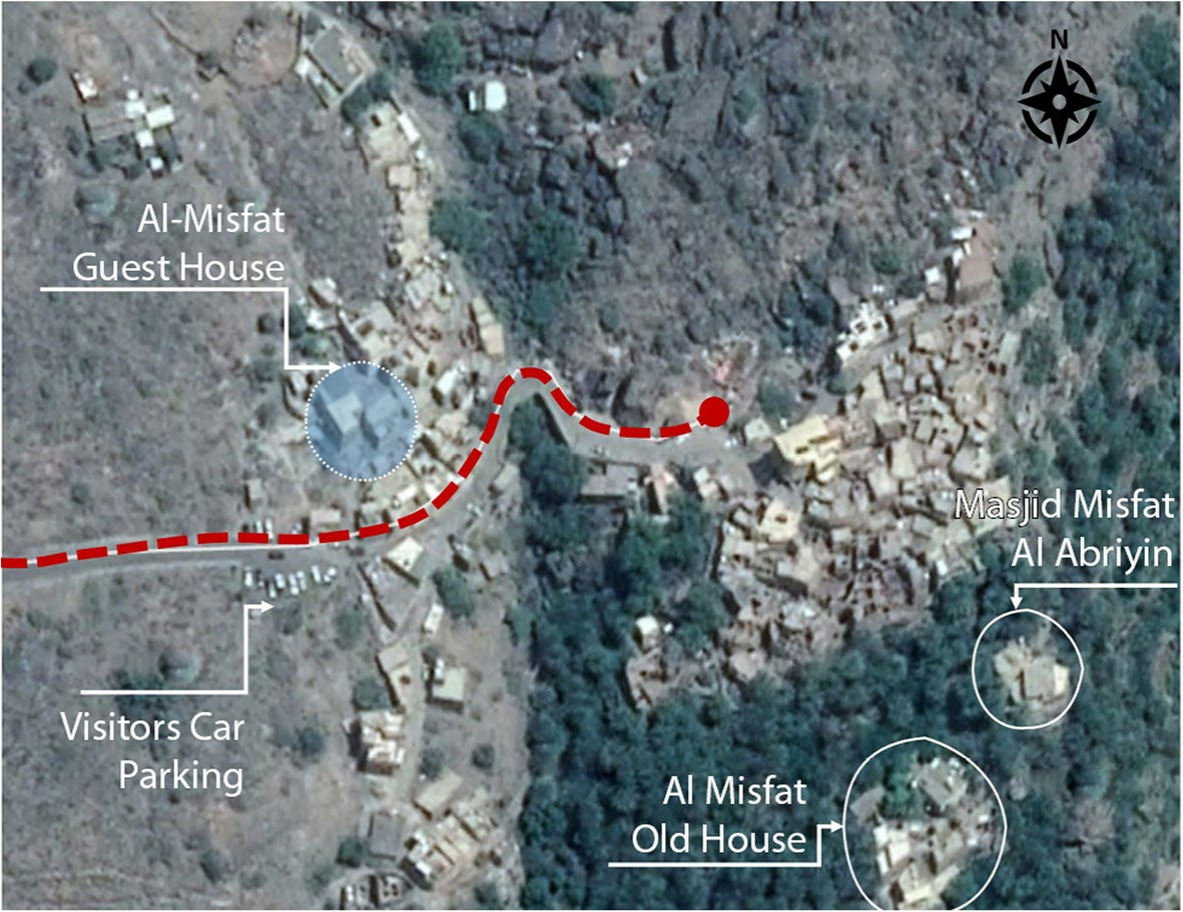
Misfat al-Abriyin, 2021 (Source: Google satellite image, enhanced by the author)

Misfat al-Abriyin is situated at 2587080.21 N, 544018.32E and occupies the steep banks located above the convergence of two wadi systems at the intersection of Balad-Seet in wadi Bani-Awf and Al-Hamra. The nucleus of the settlement grew above falaj and the basin (leguel) that irrigated its farmlands, which were installed on fortified terraces filled with arable land (Fig. 4).

The settlement is accessed through narrow, convoluted alleys, intermittently occupied by first floor rooms connecting two houses, which generate shaded passages similar to the sabats of North African and Middle Eastern medinas. The entrance of the village is also roofed by such structures and furnished with built-in benches. Such equipped entrances, commonly called ‘sabah’, are characteristic of Omani-fortified settlements and buildings (Benkari 2017b). Structures made of mountain rocks and earthen bricks wrap around the mountainside with planted terraces. Dwellings are at the top, while the farms occupy the slopes. The buildings’ walls are made of local stones and sarooj. The terraced roofs are supported by wooden beams cut from date palms and from locally available wild olive trees, on which rest smaller palm trunks that are covered with palm ribs and thick layers of loam (Bandyopadhyay and Quattrone 2016, 23,25). In terms of height, these houses can be up to two floors, with accessible terraces. The latter were often used as sleeping places during summer nights, in addition to their use for drying dates and other agricultural products at harvest. The Ministry of Heritage and Tourism conducted an initial restoration of a few houses at the entrance of the settlement, which then remained empty and closed for years following the completion of this operation. This is a typical result of such efforts; it represents a dysfunctional system of centralised decision-making, in which local communities do not adhere to the decisions applied through a top-down process.

**Fig. 5:**
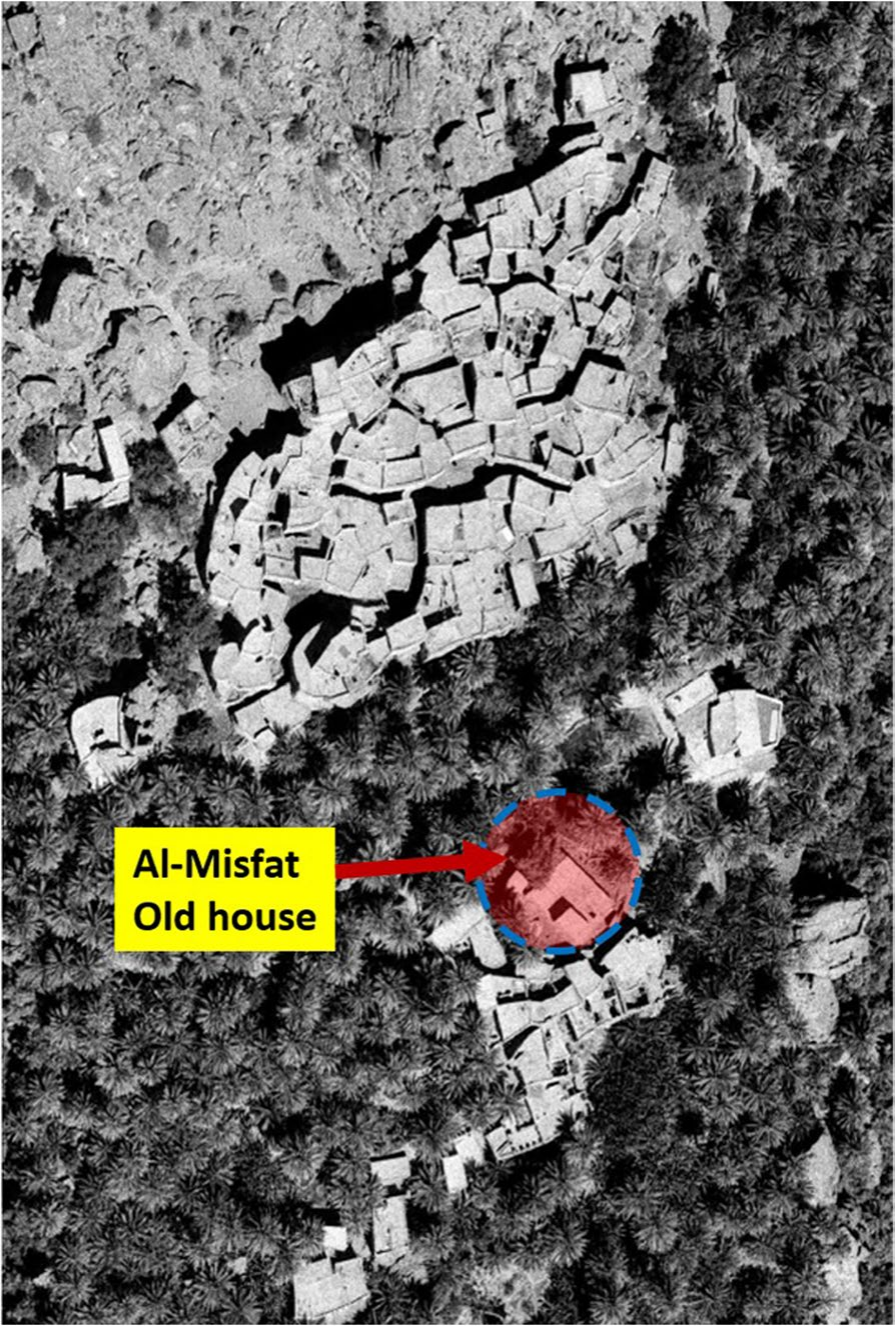
Al-Misfat Old House location (Source: Bandyopadhyay, Soumyen, and Giamila Quattrone 2016, enhanced by the author)

**Fig. 6:**
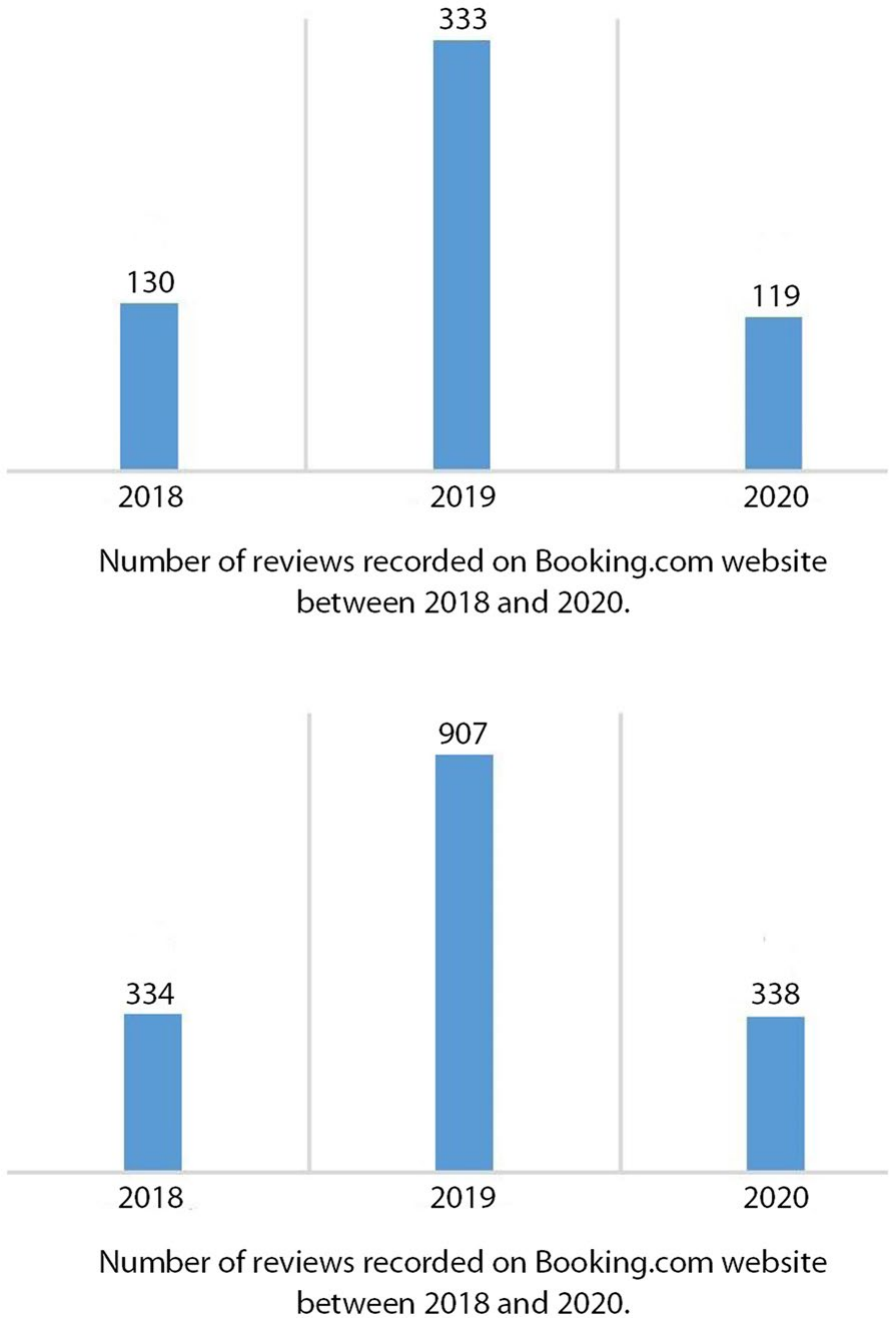
Number of customers in Misfat Old House (2018–2020) (Source: the author, based on Booking.com reviews)

The relevant initiative of Misfat Al-Abriyin is an enlarged family project led by Ahmad al-Abri, his five brothers and their children.^7^ All initial investors and participants belong to the same tribe, who have inhabited the Misfat for the last four centuries (al-Abriyin). When the patriarchal house became empty after the relocation of the last grandson to a newly built house, the idea of renovating and reusing it as a guest house was proposed by Ahmad al-Abri, who had retired from the tourism sector. With the approval of all the heirs, he started the project with some of his brothers and nephews in 2006. After the renovation and transformation were complete, ‘Misfat Old House’ opened to tourists in 2008^8^ (Fig. 5). It took 2 years for different authorities to establish administrative legislation for the opening of a guest house in a vernacular settlement: ‘*There were no regulations nor procedures to allow and regulate such operations* [of renovation and adaptive reuse of private old houses for tourism purposes]. *It is our project that anticipated the promulgation of the ministerial decision about our house to be a heritage hotel*’.^9^ Since its inauguration, ‘Misfat Old House’ has attracted an increasing number of tourists. Figure 6 depicts the progress of these numbers between 2018 and 2020. Numbers were generated by the authors from the reviews recorded on Booking.com, following the process described in the methodology section above.

**Fig. 7:**
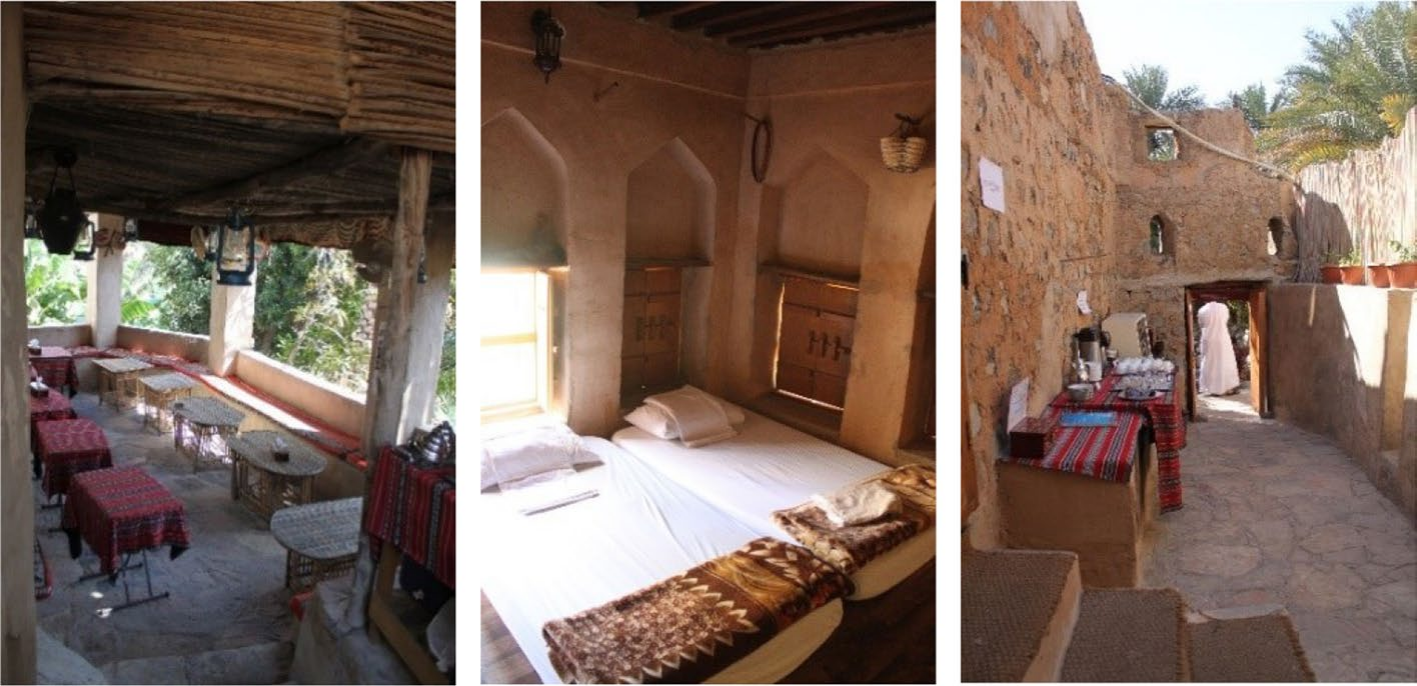
Views of the different spaces in ‘Misfat Old House’, 2016 (Source: the author)

On-site observations and interviews with the owners conveyed that many tourists book directly at the Misfat Old House,^10^ whereby those numbers are not included in the graphs presented in Fig. 6. Examination of tourists’ reviews on Booking.com also revealed that the highest numbers of reviewers were from Europe (France, 30.4%; Germany, 17%), those from Oman were in the third position (10.7%), followed by the United Arab Emirates (UAE) (8.1%). Tourists’ comments on Booking.com emphasised that the house’s traditional architecture and its location in a typical Omani village were the highlights of their experience. This has been well understood by the project owners, who started developing more activities and placing more emphasis on traditional ways of living by initiating tourists into the life of Al-Misfat. The furniture and the layout of the rooms are kept as simple and traditional as possible to cultivate this ‘exotic environment’ that it so attractive to tourists (Fig. 7).

In 2015, based on the successful initiative in Al-Misfat, a heritage management plan was developed by the former Ministry of Tourism for Al-Misfat. This plan, aimed at the sustainable development of the settlement, was elaborated with the participation of the local community, represented by the ‘Al-Misfat development cooperative’. It was executed in 2018, with funding from Bank Muscat (Bandyopadhyay and Quattrone 2016). This operation transformed some of Al-Misfat’s old houses into a coffee shop and information office at the main entrance, a restaurant serving traditional cuisine, and a traditional bakery. It has also led to the reinforcement of some collapsing gates and passages in the settlement and the addition of a new parking area.

#### Harat Al-Hamra (old settlement of Al-Hamra)

Misfat Al-Abriyin was, in fact, the second community-led initiative that invested in the built heritage of the region. The first started in the old settlement of Al-Hamra, where buildings rest on an east-west axis between 23°07′12.38″N and 23° 07′18.28″N and 57°16′38.08″E and 57°17′07.71″E. The location is approximately three kilometres south of Al-Misfat and to the south of the limestone foothill of Jabal Shams (Al-Abrī, Haitham Najeem Sulaiman: Urban pattern and architecture of traditional Omani foothill settlements: Al-Ḥamrā and Birkat Al-Mawz, unpublished thesis, 152–53; Gaube and Gangler 2012).

Archaeological investigations in the territory of Wilayat Al-Hamra have revealed that the earliest human traces date back to the early 3rd millennium B.C. (Nagieb et al. 2004; Häser 2000). Hence, hara contains traces of early Islamic buildings, dating back to the 12th century (Al-Abrī, Haitham Najeem Sulaiman: Urban pattern and architecture of traditional Omani foothill settlements: Al-Ḥamrā and Birkat Al-Mawz, unpublished thesis, 43; 138; Al-Jahwari, Nacer: Ettlement patterns, development and cultural change in northern Oman peninsula: a multi-tiered approach to the analysis of longterm settlement trends, unpublished thesis, 228). The settlement that stands today was established and inhabited by a fraction of the al-Abriyin tribe, who used to descend from Misfat al-Abriyin to plant, irrigate and harvest the oasis (Al-Abrī, Haitham Najeem Sulaiman: Urban pattern and architecture of traditional Omani foothill settlements: Al-Ḥamrā and Birkat Al-Mawz, unpublished thesis, 45). They established their first dwellings in the mid-17th century, during the reign of Imam Sultan bin Saif bin Malik Al-Ya’rubi (1649–1688), who supported the inhabitants’ efforts in widening the falaj to secure more water for their houses and agricultural lands (Al-Sulaimani et al. 2007, 3).

**Fig. 8:**
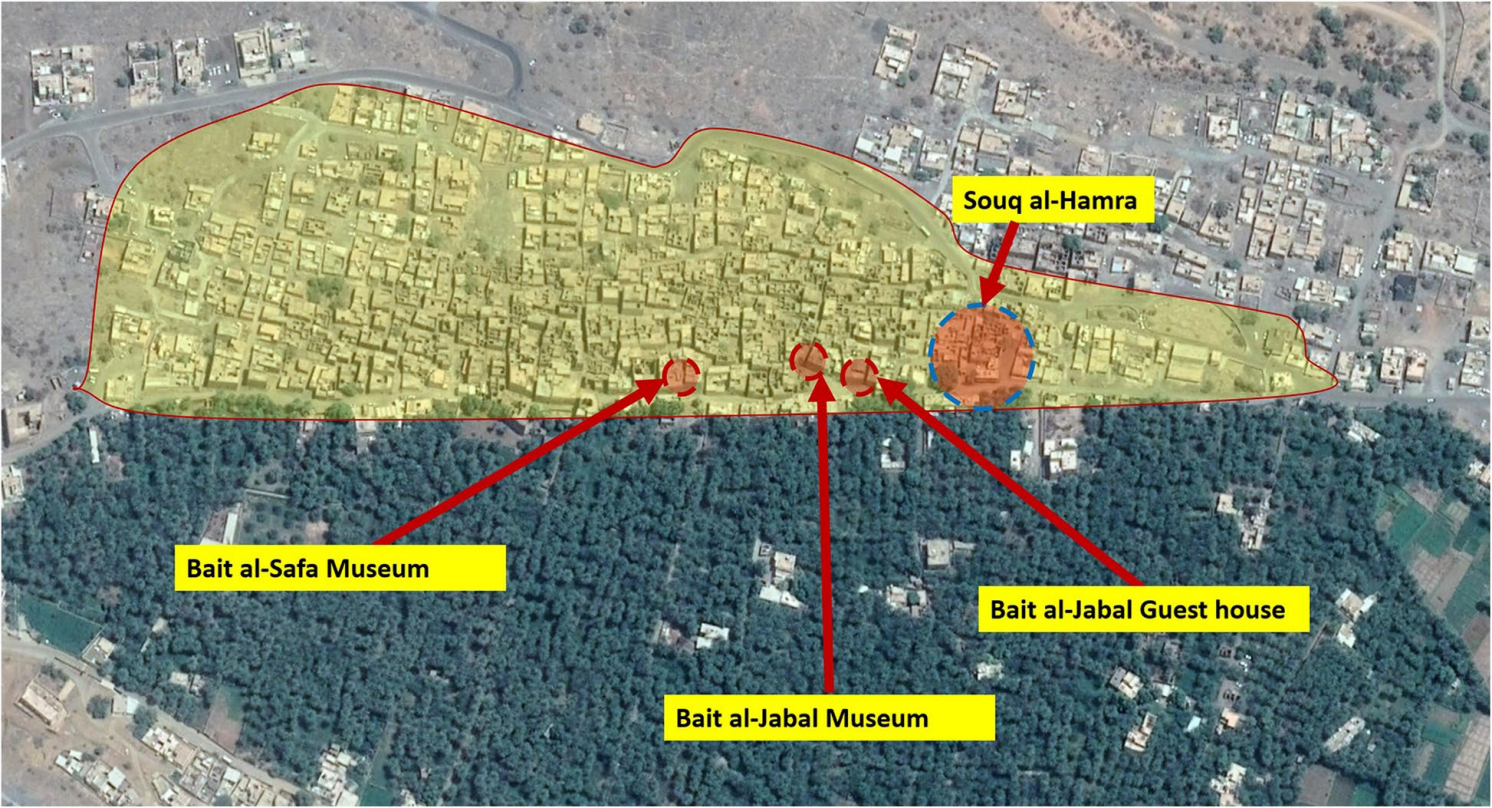
Harat Al-Hamra, Wilayat Al-Hamra, A-Dakhiliyah Region (Source: Google satellite image, enhanced by the author)

This hara is characterised by the beautiful architecture of its large houses and of the falaj that runs along the settlement’s buildings, penetrating some of them before dispersing to irrigate the agricultural lands (Fig. 8).

The settlement grew through several phases in a very organic urban pattern that adapted to the site’s profile (Al-Abrī, Haitham Najeem Sulaiman: Urban pattern and architecture of traditional Omani foothill settlements: Al-Ḥamrā and Birkat Al-Mawz, unpublished thesis, 164; Gaube and Gangler 2012). Stones of different sizes were used as foundations for walls with heights that increased according to the steepness of the slope bearing them (Al-Abrī, Haitham Najeem Sulaiman: Urban pattern and architecture of traditional Omani foothill settlements: Al-Ḥamrā and Birkat Al-Mawz, unpublished thesis, 92–93; 128). The remaining part of the 80-cm thick wall was built with sun-dried earthen bricks, and the final cladding was usually made of sarooj.

After the 1970s, native families started leaving their traditional houses in Al-Hamra to inhabit modern, concrete and air-conditioned houses. In the beginning of the 21st century, Harat Al-Hamra was almost completely deserted by its inhabitants (Al-Abrī, Haitham Najeem Sulaiman: Urban pattern and architecture of traditional Omani foothill settlements: Al-Ḥamrā and Birkat Al-Mawz, unpublished thesis, 159). Consequently, the earthen brick walls of the abandoned houses began to collapse, revealing their beautifully painted roofs and finely carved wooden doors to the few curious tourists who passed by. Such was the situation in Al-Hamra from its full evacuation in 2001 until one family decided to renovate their house, ‘Bait A-Safa’, in 2005, to make it into an ‘ecomuseum’ that depicts the traditional life of the settlement. It was the first house erected in the settlement during the mid-17th century. In fact, it is a ‘sheikhal house’ (a mansion owned by the tribe’s leader), built by Shaikh Mohammed bin Yosuf bin Talib bin Rashid Al-‘Abrī (Al-Abrī, Haitham Najeem Sulaiman: Urban pattern and architecture of traditional Omani foothill settlements: Al-Ḥamrā and Birkat Al-Mawz, unpublished thesis, 161). This house, once renovated, consistently attracted tourists to the settlement, which remained deserted of its occupants (the actual descendants of its builders).

In 2016, a local initiative to preserve the settlement and protect its buildings started to take shape. It was officially launched in 2017 as ‘The initiative of Al-Hamra’.^11^ The leaders of this initiative had a more inclusive vision about built heritage, using a holistic approach to preserve the settlement by raising awareness among local populations. Not only do they continue to renovate some houses and make them into small heritage hotels, but they regularly organise religious and civic celebrations, social gatherings and competitions for children. The latter are used to educate youngsters about the history of this settlement, its houses, mosques, and madrasas, and the scholars who lived in it. Such events are also meant to raise awareness among the foreign workers who live in the old houses of Al-Hamra, conveying the importance of its architecture. Bait A-Safa was restored for a second time with the help of this initiative, which was also used to open a heritage hotel in the settlement (Bait Al-Jabal) (Fig. 8). This hotel is composed of authentic earthen houses, which were restored with local materials through local expertise via the oversight of a native descendant from master builders.^12^

#### Harat al-‘Aqr

**Fig. 9:**
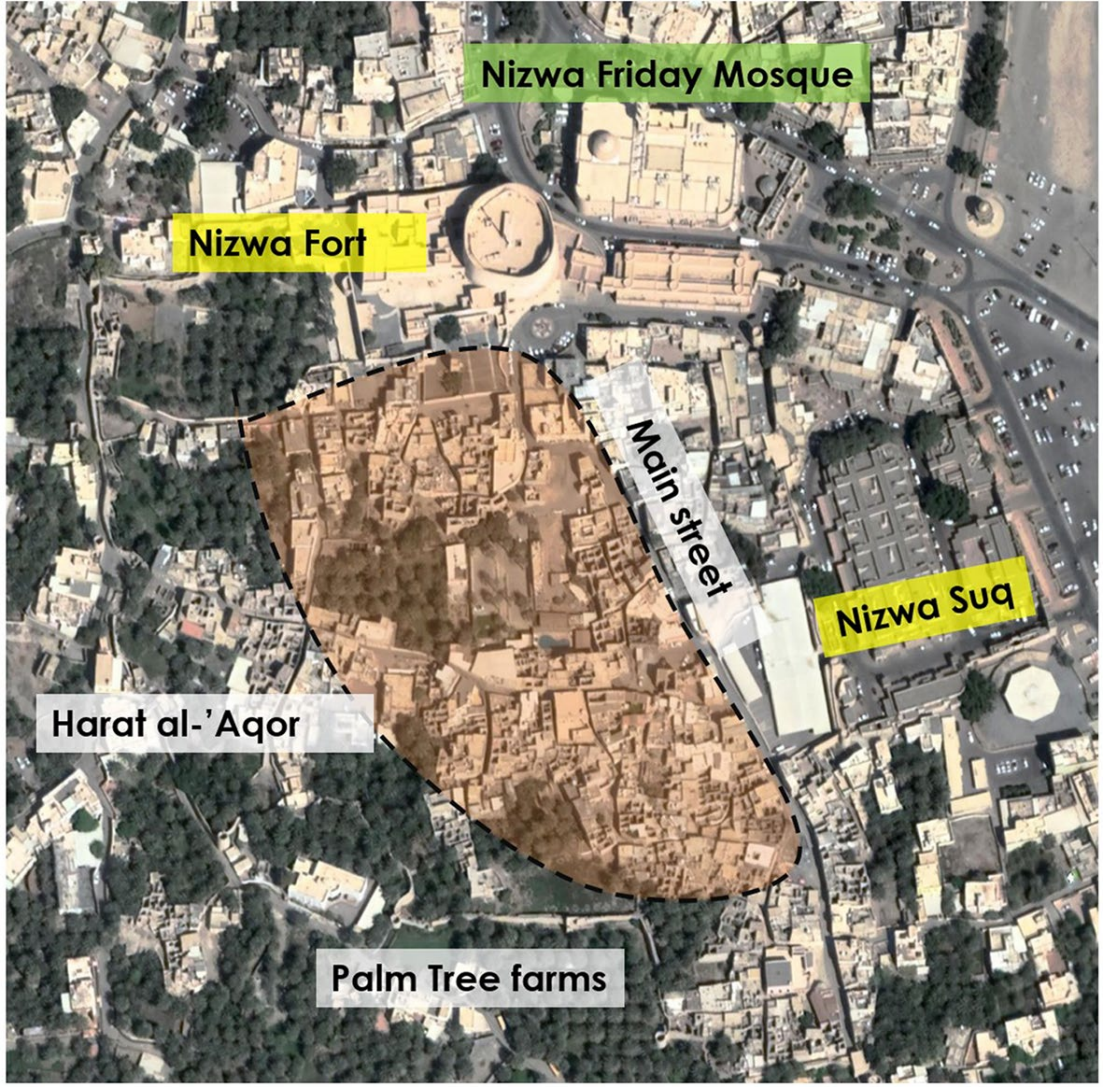
Harat al-‘Aqr, Wilayat Nizwa, A-Dakhiliyah Region (Source: Google satellite image, enhanced by the author)

The third case study is in Harat al-ʿAqr, which is contiguous with the famous fort of Nizwa. This city was an important capital and centre of power during the successive imamates who were important for the history of the interior region (A-Dakhiliyah), the most significant of which was the al-Ya’ariba Imamate (16th–mid-18th century). Nizwa and its oasis are located approximately 175 km southwest of Muscat. Bordered to the north by Jabal al-Akhdar, it rests southeast of al-Hamra. The oasis of Nizwa evolved around the confluence of Wadi al-Abyadh and Wadi Kalbu, which divided it into alaya (upper) and sufala (lower) sections. Upper Nizwa contains the ancient settlement of Samad al Kindi, while lower Nizwa contains Harat al-‘Aqr and Harat Sa’al. Al-‘Aqr is located at the southern edge of Wadi Kalbu, near its confluence with Wadi al-Abyadh. Given its prominent 17th century tower, its suq and its Friday mosque, this settlement was the core of the whole ‘oasis city’ of Nizwa (Fig. 9). Pandyopadhyay proposed a hypothetical explanation for the historical development of al-‘Aqr’s urban layout whereby the settlement’s core evolved around the Friday mosque (Bandyopadhyay 2005a, 28). The latter, which seems to have been built on a pre-Islamic structure (Bandyopadhyay 2005a, 35, note 12), was extensively surveyed before its reconstruction in the architectural style common to the ‘Sultan Qaboos great mosques’ in Oman. Based on the consulted literature, one of the oldest mosques still standing in Harat al-‘Aqor (Masjid A-Shawadhna), dates back to the 13th century, perhaps even earlier (Damluji 1998). The earliest confirmed date of 1529 CE is marked on its carved mihrab (Kervran and Bernard 1996; Baldissera 1994; Costa 2001). However, this mosque could be even older if one believes a local story about its second mihrab, which is said to have been built in the early decades of the 7th century, when Muslims used to pray facing Jerusalem (Baldissera 1994, 51; Bandyopadhyay 2005a, 28).^13^ With its falaj irrigating the palm gardens and passing through and under its dwellings, Harat al-‘Aqr presents a peculiarly dense urban fabric that is interwoven with palm gardens (Fig. 9). This layout is different from the common type of settlements in the interior region of Oman (Bandyopadhyay 2005a, 33), where the dwellings expand along the edges of arable land, similar to Harat al-Hamra, as described above.

**Fig. 10:**
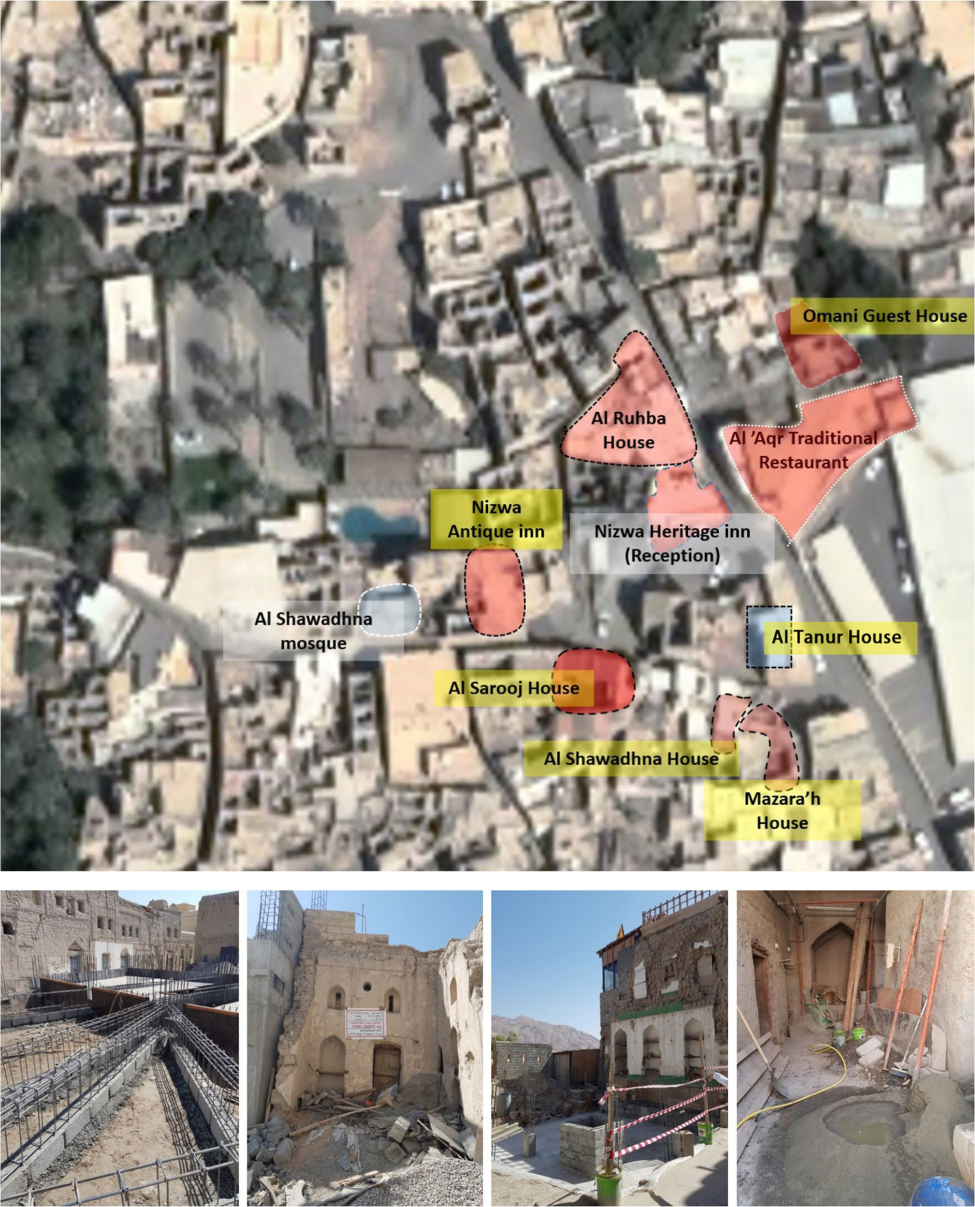
Location and photos of the ongoing constructions in Harat al ‘Aqr (March 2021) (Source: Google satellite image, enhanced by the author)

The vernacular settlement of al-‘Aqr experienced a scenario similar to the previously discussed settlements. Despite its social, political and historical importance, this hara was progressively deserted beginning in the late 1970s, and most of its houses were either rebuilt in cement or left to decay until very recently. To refurbish and rebuild many ruinous dwellings in the vernacular style to repurpose them as guest houses, a company (Bawareq Nizwa International) was founded in 2015–16 by Omani investors, some of whom were originally from the settlement.^14^ Six dwellings were converted into the first hotel (Nizwa Heritage Inn) in Harat al-‘Aqor, which was inaugurated in late 2018, offering 22 rooms, a reception office and a restaurant with local cuisine (Fig. 10). To assist the community, the company performed other small upgrades in the neighbourhood, such as preparing adequate parking for residents and visitors and installing a sewage system to accommodate the whole neighbourhood.^15^ Since its launch in 2018, the hotel has been thriving, despite a noticeable set back in 2020 due to the COVID-19 pandemic. Today, 4 years after the first hotel’s inauguration, the company manages an additional 19 dwellings that are dedicated to its extension or to becoming part of other heritage hotels in al-‘Aqr, most of which are managed by Bawareq Nizwa International.^16^ Based on the author’s site observations in March 2021, there are further ongoing developments, including more hotels and guest houses, which are being prepared for after the end of the COVID-19 pandemic^17^ (Fig. 10).

## Discussion of the studied cases

### New dynamics for the integration of vernacular heritage into local development

The built heritage in Oman presents an unquestionable potential for a high quality of cultural tourism (Buerkert et al. 2010; Benkari, Naima, and Alya Al-Hashim: Omani traditional houses and settlements: a new options for developing a sustainable tourism, unpublished). The best way to reach this ultimate goal is for the local communities to control the flow of such tourism. The three initiatives presented in this paper are all, more or less, recent and aim to renovate traditional houses, revive settlements and provide an authentic experience to visitors: an experience that reflects the genuine attachment of a community to its heritage, its wish to preserve and revive its traditions and its efforts to restore its dignity. Moreover, local communities aim to share their built heritage with visitors to generate a sustainable income for their local populations and settlements.

An important common characteristic of the three case studies is the focal role of a local community in a kind of ‘bottom-up’ management of its built heritage. Decisions are discussed at the level of a community and then shared with its representatives who convey them to the local government. It is worth mentioning that since 2016, there has been strong cooperation between representatives of the government, public investors and local communities to encourage such initiatives in this field. This phenomenon can be considered a sort of ‘vertical cooperation’ between Oman’s centres of decision making and local communities regarding vernacular heritage conservation and management.

In parallel to this ‘bottom-up’ process, a ‘horizontal cooperation’ is also in action between the different initiatives described above and has involved other groups in Oman. Our investigation reveals that the community of Misfat Al-Abriyin shares its pioneering experience with the people of Al-Hamra and al-‘Aqr. Their community representatives meet regularly to discuss their experiences and share their concerns and future projects.^18^ This very interesting ‘horizontal cooperation’ is not new to Omani society, which gives the highest importance to mutual consultation and consensus building. Historically, Omanis maintained very close ties between their tribes and communities and collectively discussed all important matters in their settlements. This highly communicative society developed a specific space, called a ‘sabla’,^19^ dedicated to this activity in their traditional settlements. The sabla has persisted as a communal space in the post-1970 neighbourhoods, even with the proliferation of social media and faster, virtual forms of communication among Omanis today. Notably, all these initiatives and projects heavily rely on these new communication platforms to showcase their progress and advertise their services. They also regularly seek national and regional media coverage. The ‘Misfat Old House’ was even featured in a special programme on the Al-Jazeera channel (Jazeera Documentary 2017). The success of this initiative has become so resonant that other governorates have requested its leaders to host workshops and debates with local communities to motivate them and guide them towards replicating this initiative in their own vernacular settlements.^20^

The encouraging aspect of these initiatives stems from their immediate effect on the physical regeneration of settlements and the strengthening of the social cohesion between the different social actors in these areas. Furthermore, such initiatives generate increasing interest in and awareness of Omani vernacular heritage among residents and tourists, including the revivals of previously lost craftsmanship in the field of construction or of cultural activities, such as those proposed in ecomuseums. Additionally, these initiatives have clearly and significantly revived the real estate markets in the studied settlements, where old earthen houses are now being sold at twice their initial price.^21^ They have also helped energize some economic sectors in the studied settlements through new food and beverage shops and have even increased demand for architects and engineers in the fields of restoration, local material production and traditional construction.

Finally, the restoration of the buildings in the three case studies is mainly completed through local efforts, local funds and local materials. This keeps the renovation costs relatively low compared to similar operations made by the MHT through national and international consultants and contractors. These cost-effective, income-generating initiatives have encouraged more people to renovate their old houses. However, one shortcoming of this procedure is that it often involves nonqualified builders and contractors, which can result in a loss of architectural identity and create serious structural and safety threats.

### Possible threats and proposed solutions

Only recently have privately owned traditional houses been included in the registration activities of the Ministry of Heritage and Tourism (Benkari, Naima, Salim Woaud Al-Araimi, and Khalifa Khalsa Al-Salmi. 2021: Oman heritage policy and its applications in the built heritage protection, under press). To date, the restoration and renovation operations of vernacular houses have been regulated as if they were new constructions. With little consideration of the authenticity of the final product, the municipalities responsible for issuing such building permits proceed on a case-by-case basis.^22^ In addition, the MHT has, thankfully, documented some endangered settlements and their buildings, yet as of now it has not developed any management or land use plans regarding these settlements. The conservation and management plan of the UNESCO-listed Bahla Oasis and fort is the first and only plan adopted thus far (Ministerial Decision No. 81/ 2019). Such plans could guide local initiatives regarding their land uses or the building regulations in traditional settlements (limits of height, materials, architectural typologies, street widths, openings, etc.). Initiatives such as those presented in this paper are expected to multiply, especially following their initial successes. Since MHT is solely responsible for the delivery of permits to open guest houses within heritage buildings, there is no coordination with the municipalities who are in charge of delivering all types of construction permits, including reconstruction or restoration works. Both the MHT and the municipalities deliver their authorisations for punctual interventions and in the absence of any kind of regulations for the preservation of the authenticity or the coherent land use in the historic settlements.. There is an urgent need for local regulations and guidelines to ensure the sustainability of such operations and the settlements they take place in.

Furthermore, the community-led initiatives studied here are characterised by the limited involvement of architects, structural engineers and qualified technicians and builders with expertise in the restoration of vernacular earthen and stone architecture. This situation compromises building safety during and after structural restorations and affects dwellings’ spatial typologies and settlements’ original urban layouts. The involvement of communities in the renovation and adaptive reuse of their built heritage could be considered an opportunity to balance the roles of politicians, experts and owners in such operations. However, communities’ limited awareness regarding the value of their vernacular heritage and sometimes, their ignorance of its components and unique characteristics, represents a real danger to the continuity of this heritage for future generations. If standardised, mass-produced beddings are used to furnish traditional rooms made for sitting and sleeping on the ground, and if large parts of houses are completely erased and centenary palm trees are uprooted to create a hotel swimming pool or a parking lot, then it is legitimate to question the viability of such initiatives.

**Fig. 11:**
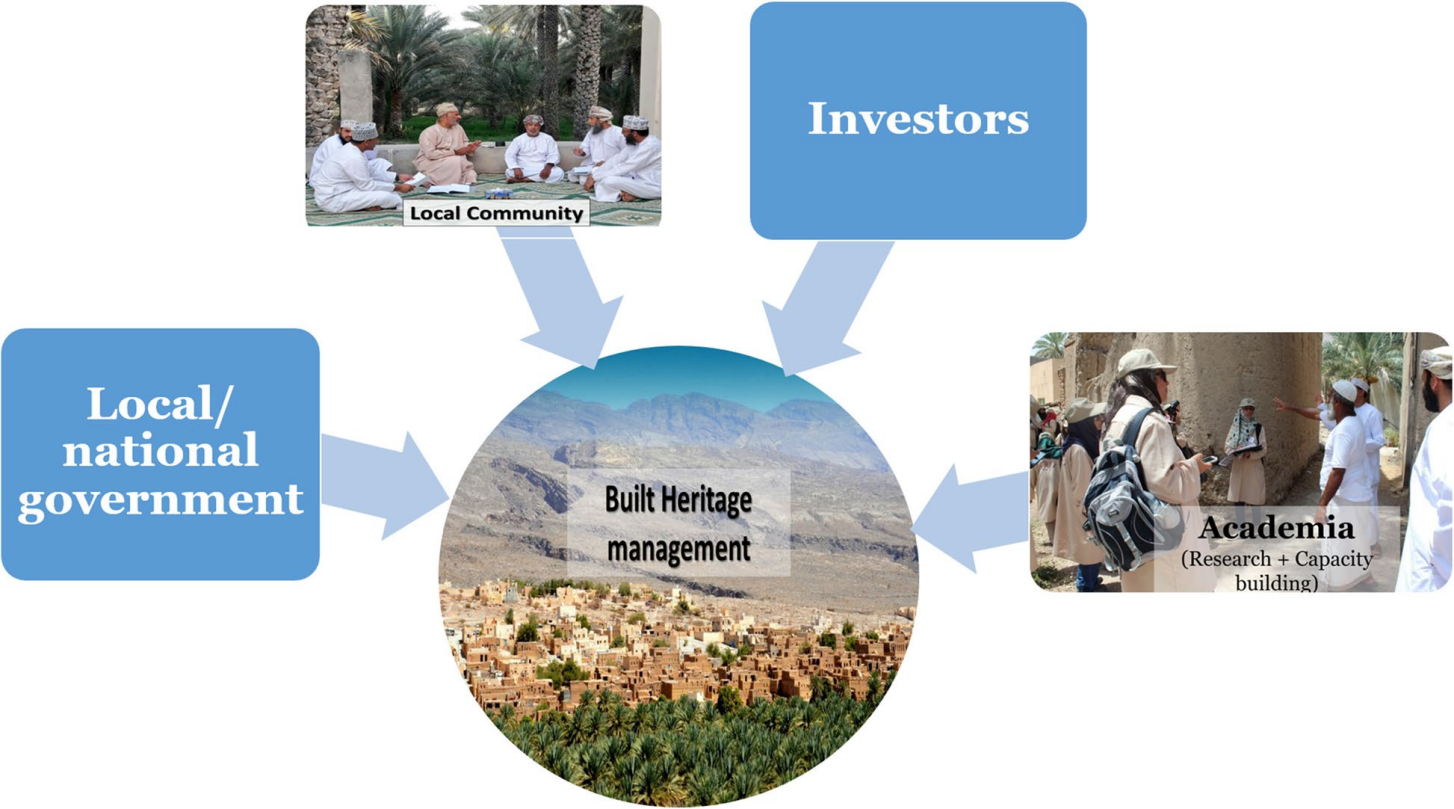
The four main actors involved in vernacular heritage renovation and reuse (Source: the author)

To help these enterprises become more culturally sensitive, socially inclusive and economically profitable, there is a need to strengthen the cooperation between the ‘quartet’ composed of local and national governments, local communities, private investors and academia to foster research, development and capacity building (De Filippi 2005, 3; Lenik 2013, 9,10; Chirikure and Pwiti 2008; Klinger 2020, 478–79; Benkari 2019) (Fig. 11). These four poles should work collectively to properly manage this heritage and give it a constructive and productive dynamic. Indeed, it is crucial to raise awareness about the value of this vernacular heritage and the importance of its regeneration for and its integration into the economic and urban development of Oman’s regions. This increased awareness would not only help secure technical support and expertise for these restorations, but also provide guidelines and master plans for the regeneration and reuse of these settlements. Such actions should be planned and proactive rather than reactive (Benkari 2019). Moreover, an efficient collaborative system must be established in which all actors contribute together to these operations for optimum outcomes for Oman’s built heritage, local communities and visitors alike.

Finally, investment in creating professionals who are skilled in vernacular architecture restoration and local material production is essential to make these operations safer and more affordable. Such professionals would also contribute to the progressive transformation of the construction sector in Oman by encouraging the use of more sustainable construction materials and methods while providing new job opportunities.

## Conclusion

The present study examined the processes and modes of vernacular heritage restoration and adaptive reuse by local communities. It has examined case studies of community-led initiatives in the rehabilitation of traditional dwellings in Oman. These are unique instances in which such operations were decided, programmed, funded, and realised by involved community members. The government was relied upon only to provide legal authorisations. Such operations, initiated in the second decade of the 2000s, are proving successful thus far, despite the drop in tourism caused by the COVID-19 pandemic. Beginning in 2016, the primary data for this research were collected from multiple site visits and observations, semi structured interviews with stakeholders, and collections of online visitor reviews.

The research showed that vernacular heritage in Oman has been somewhat lightly addressed by heritage protection and management legislation; a top-down process with decisions centralised in Muscat. This framework has negatively affected the operations of rehabilitation and reuse of heritage buildings and settlements in the country.

Through the cases discussed in this paper, it is clear that new dynamics are impacting the regeneration and adaptive reuse of vernacular dwellings. Local communities have developed a kind of ‘bottom-up’ management of their built heritage. These initiatives are gradually consolidating into a ‘vertical cooperation’ between the centres of decision making and local communities. Additionally, a ‘horizontal cooperation’ between the communities studied in this research has been demonstrated. These privately funded operations have multiplied and spread in recent years due to thriving cultural tourism in Oman.

However, such initiatives continue to be performed by nonqualified builders, and there is little to no involvement from professionals or experts in restoration. This may result in an architectural identity loss or serious structural and safety threats. In addition, no official management or land use plans have been developed for vernacular settlement rehabilitation. The authenticity and coherence of an urban typology in these settlements are not monitored by municipalities, and restorations are regulated as though they are new constructions.

The success of any community-led initiative depends largely on the existence of a proper legal framework and adequate support from governmental policies, experts and professionals in the field. Therefore, there is a need to strengthen the cooperation between the quartet of governments, local communities, investors and academia. It is also necessary to invest in the creation of skilled professionals in vernacular architecture restoration to make such operations safer, more affordable and more sustainable.

Despite insufficient data on the effects these operations have had on the urban fabrics of the studied settlements, it has been observed that these cost-effective, income generating initiatives encourage more people to replicate them. These punctual renovations have significantly improved liveability and have energised the real estate markets in the studied settlements. They have also helped revive some professions and economic sectors in or around the rehabilitated areas.

The findings of the present research demonstrate how involving local communities is an essential condition for the success and sustainability of any built heritage management operation. This involvement is an opportunity to balance the roles of politicians, investors, residents and experts. The processes and models discussed in this research could be transferred to other international regions with similar conditions regarding their vernacular heritages, administrative frameworks and socioeconomic dynamics.

Finally, this paper highlights a phenomenon in the making and cannot accurately speculate on its possible future implications. There is a need for further study of the situation to observe its expected expansion into new geographic areas and any transformation(s) in its modes of action.

## Data Availability

The datasets used and analysed during the current study are available from the corresponding author on reasonable request.

## Abbreviations

ArCHIAM: Centre for the Study of Architecture and Cultural Heritage of India, Arabia and the Maghreb
GCC states: Gulf Cooperation Council states
MHT: Ministry of Heritage and Tourism
TANFEEDH: The National Program for Enhancing Economic Diversification
UAE: Unite Arab Emirates
UNESCO: United Nations Educational, Scientific and Cultural Organisation

## Footnotes

1 Omran website: www.omran.om

2 Asaas website: www.asaas.om

3 A falaj (Pl.: aflaj) is a traditionally dug and built water channel to bring water, through very precisely calculated directions, to the surface of the oasis or the settlement, either from the water sources in the mountains or from underneath the ground.

4 ‘*Sarooj is an ancient lime mortar, part of the group of hydraulic mortars, which reaches its best performance in conditions of more or less high moisture*’ (Meddah et al. 2020, 1).

5 Interviews in al-Hamra (Bait al-Jabal guest house), on 21 April 2018, with Mr. Malik b. Hilal b. Mohamed al-Abri, lecturer at the University of Nizwa, former member of the Shura Council, and president of al-Hamra local initiative: ‘al-Mubadara al-Ahliya li Awfiya Harat al-Hamra’, and Mr. Jaber b. Mubarak al-Abri, contractor in restoration, director of the Jabal Heritage hotel in al-Hamra and actual president of al-Hamra local initiative.

6 Interview on 5 February 2016, in Misfat al-‘Abriyin, with Mr. Nayef al-Abri (Responsible for bookings and communication in ‘Misfah Old House’). He is also the nephew of Mr. Ahmad al-Abri, the main partner and initiator of the project.

7 Interview with Nayef al-Abri, in Misfat al-‘Abriyin, on 5 February 2016.

8 Ibid.

9 Ibid. This ministerial decision was issued by the Ministry of Commerce for this specific house. Indeed, private monuments were not included in the list of protected sites and buildings until 2017. The Cultural Heritage Law 35/2019 addressed this category of buildings and further regulated their listing criteria and the process (Benkari, Naima, Salim Woaud Al-Araimi, and Khalifa Khalsa Al-Salmi: Oman heritage policy and its applications in the built heritage protection, under press).

10 Interview with Nayef al-Abri on 5 February 2016.

11 Interview with Mr. Malik b. Hilal b. Mohamed al-Abri, on 21 April 2018, in al-Hamra (Bait al-Jabal guest house).

12 Interview with Mr. Jaber b. Mubarak al-Abri, on 21 April 2018, in al-Hamra (Bait al-Jabal guest house).

13 Interview with Mr. Sulaiman b. Mohamed b. Sulaiman al-Sulaimani, member of the administrative board of the Bawareq Company, Director of the new ‘al-Bustan’ Hotel in Harat al-‘Aqr (12 February 2021). The author has also observed the presence of this second mihrab within a much smaller niche in a corner of the prayer hall in the northern wall.

14 Interview with Mr. Zahir b. Sulaiman b. Issa al-Zakwani, General Manager of Bawareq Nizwa International for Investment (23 July 2018, Harat al-‘Aqr).

15 Ibid.

16 This includes hotels such as: al-Bustan, a-Rahba, al-Hosn, a-Tanur, al-Falaj. Interview with Mr. Sulaiman b. Mohamed b. Sulaiman al-Sulaimani on 12 February 2021, and follow up updates with him and Mr. Zaher al-Zakwani on 25 March 2021.

17 Ibid.

18 Interview with Mr. Malik b. Hilal b. Mohamed al-Abri on 21 April 2018 and Mr. Zahir b. Sulaiman b. Issa al-Zakwani on 23 July 2018.

19 ‘*The sabla, symbol of the ‘democracy’ and hospitality of the Omani traditional society is an essential component in the vernacular settlements*’ (Benkari 2017b).

20 Reported to me by H.E. Sayed Ibrahim b. Said al-Boussaidi Minister-Governor of Musandam in a meeting on 5 January 2021.

21 Interview with Mr. Sulaiman b. Mohamed b. Sulaiman al-Sulaimani, Director of the new Hotel ‘al-Bustan’ in Harat al-‘Aqr (12 February 2021).

22 Ibid
